# Follicular unit grafting in chronic ulcers: a valuable technique for integrated management^☆^

**DOI:** 10.1016/j.abd.2023.08.012

**Published:** 2024-03-22

**Authors:** Anahi Belatti, Florencia Bertarini, Virginia Pombo, Luis Mazzuoccolo, Damian Ferrario

**Affiliations:** Department of Dermatology, Hospital Italiano de Buenos Aires, Buenos Aires, Argentina

**Keywords:** Follicular unit, Hair follicle stem cells, Hair transplantation, Punch grafting, Skin grafting, Venous leg ulcer, Wound healing

## Abstract

Chronic ulcers significantly affect the quality of life of patients and impose a high cost on the healthcare system. The therapeutic management should be comprehensive, taking into consideration the etiological diagnosis of the wound and the characteristics of the wound bed when deciding on a therapeutic proposal appropriate to the healing phase, correcting factors that delay healing. During the epithelialization phase, repair techniques with grafts are recommended to shorten re-epithelialization time, improve the quality of scar tissue, and achieve adequate pain management. Currently, due to the reported benefits of skin appendages, the technique of follicular unit auto-grafting obtained with a scalp punch is among the chosen strategies for wound repair. This is a minimally invasive, outpatient practice, whose technique has advantages over the donor site, patients recovery and well-being.

## Introduction

The therapeutic management for the healing of chronic wounds is broad, with a conservative treatment based on moist wound healing, which includes multiple options that are not always satisfactory.^1^, ^2^ For this reason, advanced healing options must be sought, including surgical techniques, to shorten the re-epithelialization time. Skin grafts are classified according to their origin and the technique used: autograft when taken from the patient or after growing the patient's cells, allograft taken from other human sources and xenograft usually taken from pigs. In chronic wounds, autografts are frequently used because they are a simple outpatient practice with good results.^3^ They are classified according to their thickness, into split-thickness grafts (when they include the epidermis and part of the dermis with a thickness of 0.2‒0.4 mm) or full-thickness skin grafts (complete epidermis-dermis and skin appendages = 0.6 mm thickness or more).^4^ Two main techniques are described for their obtainment: pinch and punch or punch biopsy.^5^, ^6^

Historically, donor sites for autografts have been regions such as the thigh or abdomen due to the similarity of skin characteristics to the recipient site and ease of healing.^7^

Currently, due to the benefits mentioned and highlighted by skin appendages, the technique of follicular unit auto-grafting obtained from the scalp punch is among the chosen strategies for the management of recalcitrant ulcers with poor response to standard treatments, showing a full recovery in a reasonable time of the donor site, requiring minimal care, thus alleviating, and providing greater well-being to the patient.^8^

## Purpose of the article

The authors aim to accurately convey the protocol for implementing this novel technique in wound management developed in the Wound Healing Section of the Dermatology Department of Hospital Italiano de Buenos Aires. The authors will provide a brief overview of the demonstrated benefits of skin appendages, patient selection, and preparation, conditioning of the wound to be grafted, technique used during the procedure, and follow-up. Based on conclusions and literature review, the authors will show the advantages identified so far from previous experience.

## Demonstrated benefits of follicular units

The benefits of using scalp skin as a donor area were first described by Crawford in 1964 in the context of a patient with extensive burns (60% body surface area) in which there were no possible donor sites, and the possibility of using scalp skin was considered assuming that the existence of hair follicles and their depth would promote healing.^9^

Currently, it is known that obtaining follicular units from the occipital scalp region provides benefits that are related to the obtaining of pluripotent mesenchymal and epithelial stem cells from anagen-phase hair follicles and the release of leptin (a hormone with stimulating properties for healing) by dermal papillae in this context of anagen induction.^7^, ^10^, ^11^, ^12^, ^13^, ^14^

In addition to this benefit, the rapid healing of this area with imperceptible scar sequelae is added.^13^, ^15^

A prospective study carried out in 2 groups of pediatric patients with burns compared the use of 2 donor areas for grafts, thigh and scalp, the benefits of the technique, the evolution of the donor areas, and results, finding that the scalp represented a better donor area for this type of wound.^16^

In several cohort studies of patients with lower limb ulcers, better healing response has been demonstrated with follicular grafts than with conventional techniques.^17^, ^18^

In a randomized and controlled study, chronic ulcers of the lower limbs were selected, each of which was divided into 2 randomized halves to receive, the experimental half, 2 mm punch grafts harvested from the scalp, and the control half, 2 mm punch grafts of abdominal skin without visible hairs. In the comparative study, the statistically significant difference in the evolution towards the healing of the experimental half that used scalp follicular grafts could be observed.^18^

## Patient preparation for the technique

### Selection

In the authors’ experience, suitable candidates for the technique are those with ulcers that show a lack of response to other implemented techniques for advanced wound healing, with stagnation in the evolution towards healing and/or ulcers with devitalized or senescent beds.

It is important to highlight that in this type of devitalized tissue, structurally and anatomically modified by the chronicity of the wound, follicular unit grafts show an improvement in the characteristics of the scar, increasing the resistance of the new epithelium that arises from this stimulus and also providing a better microenvironment with a lower risk of recurrence.

Also eligible are patients who have good progress and wish to accelerate the healing process for complete closure of the wound, which could otherwise take several months for complete epithelialization, thereby increasing the risk of complications.

Regarding the etiology of the ulcer, the most frequent indications are those of vascular origin (venous, arterial, or mixed), but the authors have experience in other etiologies (vasculitic ulcers, inflammatory pathologies, blistering, radiodermatitis, etc.) when these are stable and under specific treatment. It should be noted that when the diagnoses correspond to atypical ulcers or the patients have comorbidities that may affect the acceptance and response to the graft (e.g., immunosuppressive treatments), it is explicitly stated when offering this technique as a therapeutic alternative, emphasizing that the response may be beneficial but more limited or slow.^19^

Ideally, the recipient area should have a granulating bed at a superficial level that would be enhanced by the stem cells of the follicles to generate foci of active epithelium. In the authors’ experience, this technique shows that patients who had halted healing, with devitalized, fibrotic beds, defined as senescent ulcers, and whose delay in epithelialization was not explained by associated complications (such as local infections, malignancy, or another unstudied etiology of ulcer), are also benefited by the stimulus provided by the follicular unit to the bed and by a phenomenon of hyperstimulation of the wound edges that accelerates epithelialization from the periphery.

### Bed preparation

There are several therapeutic options used to achieve the angiogenic stimulus of the bed that deserve a separate chapter and are not the focus of this article, but the authors can list some of those used in previous experiences: topical application of phenytoin, timolol, insulin, maltodextrin, and hydrocolloid powder, among others.^2^, ^20^, ^21^, ^22^, ^23^ Prior grafting therapies seek to stimulate the wound bed, fostering a highly receptive environment of granulation tissue for the follicular units. Through effective control of factors such as inflammation, infection, and pain, achieving a graft-ready bed within 2 to 3 months becomes attainable. Unfortunately, the treatments mostly used in practice have low-quality evidence. However, the authors use them by selecting a suitable patient.

Additionally, it is crucial to control the biofilm inherent in chronic wounds, reducing the bacterial load to avoid post-graft complications, using timely sharp debridement, washing with antiseptic solutions with active principles such as Polyhexamethylene Biguanide (PHMB) or 4% chlorhexidine, dressings with nanocrystalline silver or silver sulfadiazine dressings, or even in those patients with a history of repeated infections, instituting antibiotic prophylaxis in the days prior to and during the graft.^24^

### Pain management

In patients with chronic neuropathic pain, the baseline treatment (pregabalin, gabapentin, opioid derivatives, among others) is maintained according to individual needs to achieve mild pain scales or absence of pain, using the numeric verbal pain scale as a reference. Reinforcements may be indicated before and after the surgical technique, in a regulated and individualized manner, and short-term use of nonsteroidal anti-inflammatory drugs may be considered.^25^, ^26^, ^27^

### Surgical technique description

The technique is performed in a single surgical session and consists of grafting a certain number of hair Follicular Units (FU) from the scalp into the bed of the ulcer to be healed. The intervention is entirely performed under local and/or regional anesthesia in an outpatient surgical room.

It is important to select the patient appropriately based on their physical condition. Factors to consider include the patient's ability to mobilize (remain in dorsal and/or lateral decubitus position throughout the procedure), their psychological condition, and pain threshold (as they will receive multiple anesthesia punctures on the ulcer and scalp).

It should be noted that the patient must understand the benefits of the technique for their pathology and consider the possibility that a percentage of the grafts may not be viable and/or the possibility of complications like pain, infection, inflammation or edema, all of which are stated in the informed consent that accompanies the procedure and the prior preparation.

For better organization and optimization of the procedure, the authors recommend forming two working teams: the first team will be responsible for the ulcer to be grafted (Team 1), and the second team for the scalp which will harvest the FU (Team 2). Both teams can be composed of one or more physicians, but it is important to keep in mind that the more people performing the practice, the greater the coordination needed between them to avoid hindering their movement in the surgical room.

**Step 1: Measurement of the ulcer and calculation of the number of FU to be harvested**

**Figure 1:**
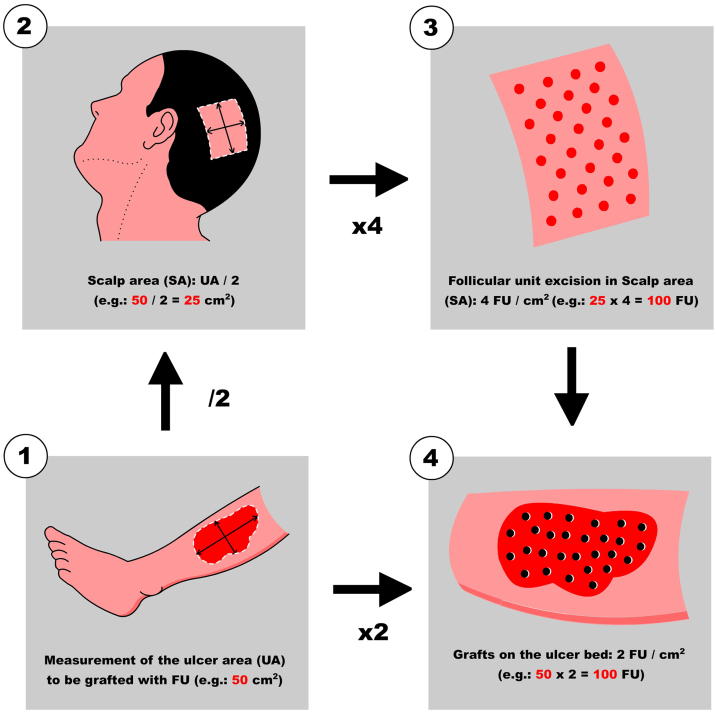
Quick view for the calculation of follicular units (FU) per ulcer area to be treated (UA, Ulcer Area; SC, Scalp Area).

First, Team 1 measures the area of the ulcer in cm^2^ (Fig. 1), and then calculates the necessary number of FU to be grafted. To simplify this calculation, the authors recommend planning a density of 2 FU/cm^2^ (1‒3 FU/cm^2^), which means that if the surface of the ulcer to be grafted is 50 cm^2^, 100 FU will need to be grafted. Based on this quantity, Team 2 calculates the surface area to be shaved in the temporoparietal region of the patient's scalp to harvest those 100 FU (Fig. 1). Based on a simplified line of reasoning, if the authors can harvest a maximum of 4 FU/cm^2^ (3‒5 FU/cm^2^) in the scalp, we should shave 25 cm^2^ (the calculation consists of dividing by “2” the surface area of the ulcer to be grafted). Subsequently, the location to be shaved (occipital, parietal) is decided based on the pattern of alopecia and/or the length of the patient's hair at the time of surgery (preferably parieto-occipital due to the higher density of anagen FU). As for the shape of the area, it can be square, rectangular, or circular. Longitudinal coronal shaves are a good option in patients with long hair, as after harvesting, the punch scars will be better hidden (as if they were curtains). The shave can be done with Mayo or Metzembaum scissors, leaving the hair length between 2 to 3 mm. If a razor is used, it should be disposable.

**2^nd^ Step: anesthesia of the ulcer along with the scalp area to be harvested**

In order to reduce the pain associated with injections of anesthetic into the periphery and ulcer bed, it is suggested to apply lidocaine gel occlusive to the surface of the ulcer 10 minutes before the procedure. Injections are performed into healthy tissue, addressing the entire circumference of the wound (Video 1 - Supplementary material).

Concurrently, anesthesia is performed on the scalp. The authors’ preference is to perform the nerve block anesthesia first, followed by tumescent anesthesia within the shaved area to harvest the hair follicles. With regard to the technique of nerve block anesthesia, this can be done in two ways: the first consists of placing it punctually on the nerve trunks (for example, the points of the occipital nerves); and the second, as a ring under the shaved area (regional nerve block). Each syringe is composed of two equal parts of 2% lidocaine and 1/100,000 epinephrine, on one hand, mixed with 2% bupivacaine.^28^ This dilution allows us to generate hemostasis during the procedure and increase the duration of anesthesia. Then, tumescent anesthesia (Video 2 - Supplementary material) is placed on the entire surface of the shaved area using a buffered anesthetic preparation (35 cubic centimeters (cc) of physiological saline, 3 ampoules of 2% lidocaine plus 1% epinephrine, and 10cc of bicarbonate), which further reduces bleeding during the procedure (mechanical compression of blood vessels), moving vascular trunks (above the galea = aponeurotic layer, richly vascularized) away from possible damage by the punch.^28^

**3^rd^ Step: Extraction of FUs from the scalp**

A 2 mm punch is used for this purpose. The technique consists of first placing the punch vertically on the skin surface and using quick circular movements with the middle and ring fingers, it is deepened until the dermis. Without removing the punch, continue obliquely parallel to the hair shaft until reaching the Subcutaneous Cellular Tissue (SCT) at 3‒4 mm depth. With the initial vertical entry and then oblique, the punch incision will leave a circular defect and not an oval shape (if the entry were inclined throughout the path). Then, with oblique and parallel deepening to the hair shafts, transection of the hair follicle is avoided, keeping the structure intact (Video 3 - Supplementary material).

One thing to keep in mind is the distribution of punctures: remember that they should be performed regularly and symmetrically (4 FUs/cm^2^), to avoid overlapping with the consequent risk of necrosis of the scalp skin.

A delicate curved clamp (jeweler) is used to harvest the FUs. With the curvature upwards, the dermis is delicately grasped, performing a longitudinal dragging maneuver to avoid damaging the FU. If the technique was correct with the punch cut, it would come out easily and the surgeon should not pull with force.

**4^th^ Step: Transport and maintenance of FUs in saline solution**

**Figure 2:**
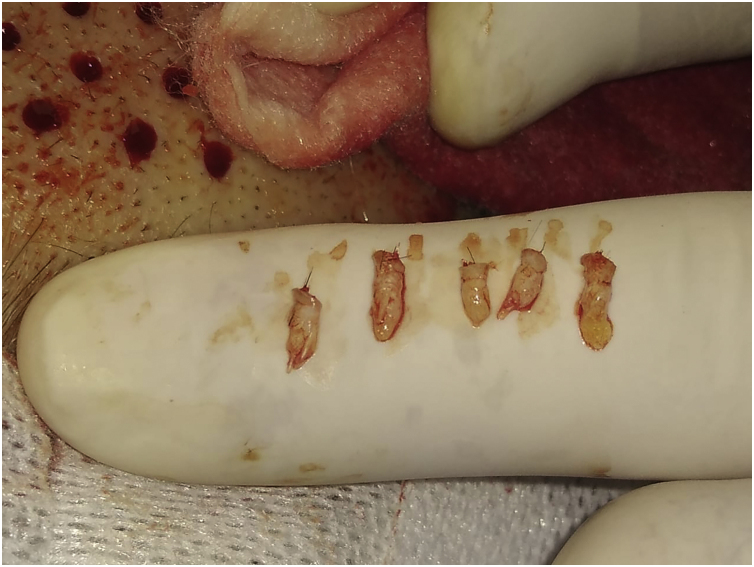
Harvested follicular units (FUs).

After extraction, the grafts are placed in a sterile container with saline solution until their subsequent implantation. The authors used a container divided into compartments in which as they are extracted, they are placed in the same compartments of 10 or 20 FUs until the necessary grafts are completed. This method allows us to avoid errors in counting the FUs that are being extracted. While the scalp is healing (Step 5), the harvested FUs are delivered to Team 1 which prepares to work on the ulcer (Fig. 2).

**5^th^ Step: Healing of punctures in the scalp**

**Figure 3:**
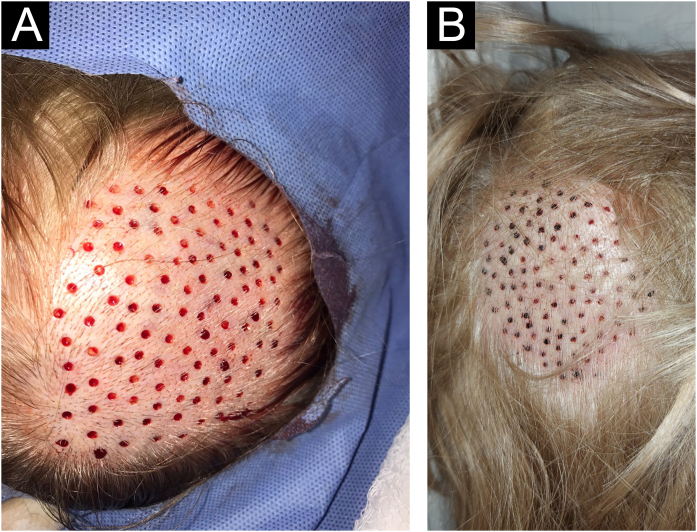
(A) Circular defects after extraction of follicular units. (B) Evolution of donor area healing at 72 hrs.

Residual millimetric circular defects (less than 4 mm) (Fig. 3A) do not require sutures, allowing healing by second intention in a variable time of 5 to 7 days, leaving only a totally imperceptible punctiform scar for the patient, which will be hidden later with the growth of the surrounding hair (Fig. 3B).

**6^th^ Step: 1.5 mm punch excision of ulcer bed tissue**

Before beginning with the pre-implantation stage, not only the quantity but also how the FUs will be distributed throughout the ulcer should be planned. As the authors mentioned previously, the implantation density should be around 1 to 3 FUs per cm^2^. The authors recommend starting with 2 FUs/cm^2^ in the first surgeries and increasing to 3 FUs/cm^2^ as the team gains more experience in the technique. The incision where the FUs will be placed is made with a 1.5 mm diameter punch vertically to 3‒4 mm depth. Then, the tissue is removed with a delicate clamp and its lower connection is cut with curved iris scissors. If this causes bleeding, continuous, and firm compression with gauze moistened with saline solution will suffice for hemostasis. In the authors’ experience, if bleeding is not too profuse, simply inserting the FU and gently compressing will generate hemostasis

**Step 7: Grafting of follicular units in the ulcer**

The insertion technique consists of taking the FU from the container with saline solution by its lower end (adipose-dermis sector) with the convexity upwards of the delicately curved tweezers (jeweler's tweezers). Then, said FU is inserted into the opening, bringing the tip of the tweezer to the bottom of the previous excision made with a 1.5 mm punch in the ulcer bed. When removing the tweezer, it is recommended to perform a gentle compression on the FU with a gauze soaked with saline solution (this suggestion allows the FU to not adhere to the gauze when compressing it in the bed) as the slightly open tweezer is vertically withdrawn. Caution must be taken during these maneuvers, avoiding the expulsion of adjacent grafts as the authors progress in the procedure (popping or jumping effect).

The graft insertion technique will also depend on the organization, experience, and criteria of the intervening medical team.

### Two techniques can be used

*Two-step technique* (**#**1): Consists of performing all the incisions with the 1.5 mm punch first and placing the grafts afterward (Video 4 - Supplementary material); the necessary punctures and distribution can be calculated more simply to perform the grafts later. It increases the speed of the procedure by performing fewer but repetitive maneuvers (punch and excision of ulcer tissue). The disadvantage is the lesser control of bleeding and the sectors to be grafted could be lost due to the retraction of the incisions and the ulcer bed. This technique could be performed by one or more doctors.

*One-step technique* (#2): This consists of performing the punches and grafts one by one until the entire surface of the ulcer is completed (stick and place technique) (Videos 5, 6 - Supplementary material). This “stick and place” technique is the one the team usually uses. The advantage is the more efficient control of bleeding in the ulcer bed due to progressive tamponade with the grafts. As the incision is made first, the exact site where the graft would go can be better visualized. The disadvantage is that having to perform multiple steps or maneuvers, greater coordination is required between the doctors on the team. The distribution and overall design of where the grafts would go may not be uniform, so the amount placed, and the amount still needed must be prospectively considered while performing this technique. Two or more doctors are required to perform this technique. The steps to be performed would be as follows:

Step 1: An incision is made with a 1.5 mm punch in the ulcer bed (Doctor 1).

Step 2: The remaining tissue is removed with delicate forceps (Doctor 2). During this step, Doctor 1 takes the graft from the container with sterile saline (or may have several grafts prepared on a gauze soaked in sterile saline) and prepares for implantation.

Step 3: The graft is implanted in the ulcer bed (Doctor 1).

Step 4: Gentle compression is applied with a moistened gauze over the grafted area (Doctor 2).

Step 5: The slightly open forceps are gently removed (Doctor 1).

**Figure 4:**
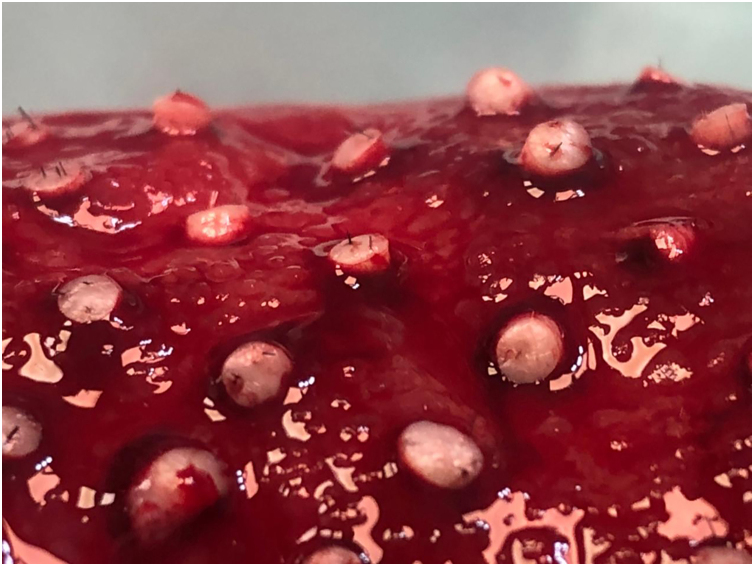
Close-up view of grafts implanted in the ulcer bed on the leg.

Step 6: The moistened gauze is removed, verifying the correct placement of the graft (Doctor 2) (Fig. 4).

**8^th^ Step: Healing of the receiving area**

**Figure 5:**
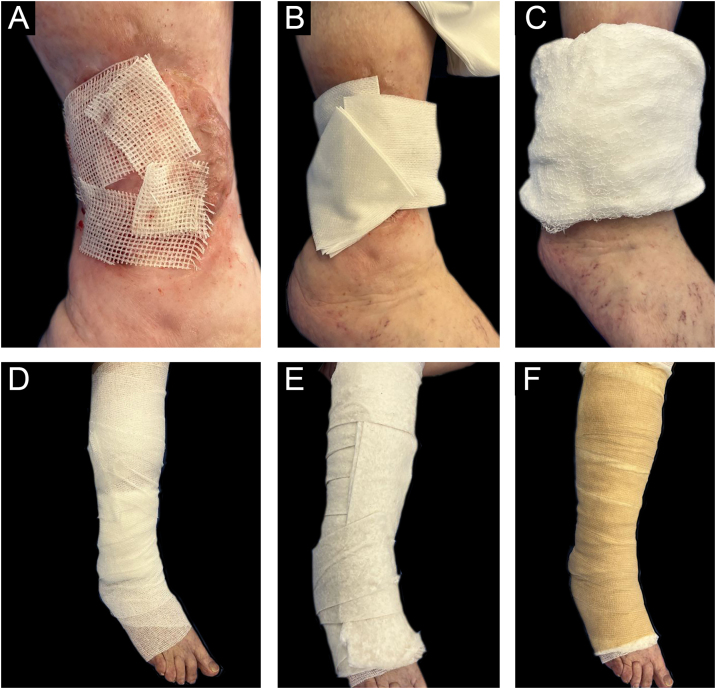
Sequence of Coverage and adapted multi-layer bandage. (A) Petrolatum-infused gauze, (B) Dry gauze, (C) Dressing, (D) Gauze bandage, (E) Ovate bandage, (F) Elastic wrap.

Once the intervention in the receiving area is completed, the grafts are covered with sterile vaseline gauze and then with a multi-layer adapted dressing consisting of microperforated gauze (non-woven gauze), dressings, cotton wool, and elastic wrap (Coban®) (Fig. 5).

**Figure 6:**
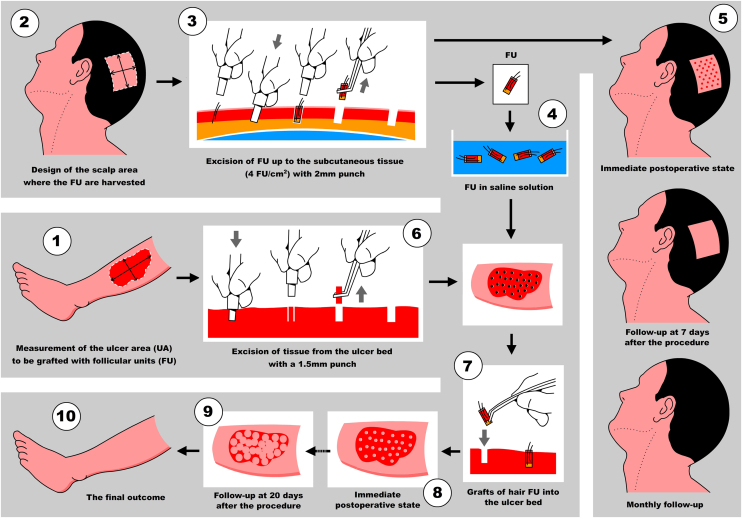
Practical illustration of the follicular units (FU) graft sequence.

You can find a schematic summary of the steps of the mentioned technique (Fig. 6).

The donor area is covered with dry gauze until the next day with instructions for dry healing with alcohol twice a day for 7 days without a secondary dressing from the second day. In the first dressings, it is important to remove the dressings slowly, after moistening them with saline solution for a few minutes, in order to prevent adhesions to the tissue and the risk of detaching the grafts. One should be attentive to the risk of infection and bleeding, as well as to an increase in pain during check-ups.

For those patients undergoing treatment with elastic compression bandages, the same protocol is maintained. There is evidence about compression therapy and its contributions to improving venous and lymphatic return, reducing edema, and improving pain, all elementary factors to favor the process of ulcer healing. Multi-layer or multi-component bandages that include 2 or more layers plus an elastic bandage have been shown to promote healing by accelerating the complete epithelialization of ulcers in less time than those who do not receive this indication.^29^, ^30^, ^31^, ^32^

Relative rest is indicated, mainly for the first 7 days (alternating periods of usual activity with rest periods of 20‒30 minutes in a supine position), then it will be up to the patient's routine. These relative rests, combined with compression bandages, favor lymphatic and venous drainage, avoiding edema, and consequently, improving the reception of grafts to the bed.^33^, ^34^

Post-grafting check-ups are performed every 72 hours for the first 7 days, then every 1‒2 weeks (depending on the complexity of the patient), removing and renewing the adapted multi-layer bandage, ideally during the first 3 weeks.

It is an outpatient procedure that does not require hospitalization or prolonged immobilization (Table 1).

**Table 1:** Post-graft care sequence.

1. Cover the grafts with petroleum jelly gauze, taking care to respect the diameter of the wound. These dressings can be vacuum-packed (such as Hidrobas® or jelonet®) or prepared with sterile gauze and solid petroleum jelly. If preparing them, excess petrolatum should be avoided, applying a thin layer of product to maintain controlled moist healing.
2. Cover the petroleum jelly gauze with dry gauze and dressings according to the diameter of the wound, giving total coverage and protection to the bed.
3. Apply an ascending bandage up to the knee with a gauze bandage to hold the dressing in place and protect the skin.
4. Apply an ovate bandage with the same technique, respecting the ascending bandage to provide coverage to the entire limb. Be careful to avoid excess folds and provide extra protection to bony areas.
5. The final step of fixation and containment is performed with Coban® wrap. This self-adhesive cohesive wrap provides fixed containment to this adapted multilayer dressing. With the same ascending technique, be very careful when using it, avoiding wrinkles or folds that could trigger inappropriate pressure on the limb and thus, a new wound.
6. Apply an elastic bandage according to previous indications.
7. Proper footwear: it is important to emphasize that for the day of the graft and afterwards, the use of footwear that allows for this bulky dressing should be advised. We suggest the use of post-surgical sandals or similar footwear with velcro adjustments, as they are adaptable and have a rubber sole. It is also important that the healthy support limb has appropriate footwear to avoid complications due to antalgic gait.

## Discussion

Regarding the morphological differences between grafts with or without FU, the benefit of using skin grafts from the scalp not only lies in the possibility of hiding the scar of the donor site and the rapid recovery of this donor area but also in the morphological differences that exist with respect to the presence of FU. Alopecic skin grafts (abdominal, gluteal, etc.) are composed of epidermis, dermis, and small fragments of subcutaneous fat without visible terminal hairs. Scalp skin grafts are composed of the epidermis, dermis, piloerector muscle, sebaceous gland, neurovascular system, and the follicular structure with the hair shaft, follicles with terminal hair, and subcutaneous fat. In this way, the authors can obtain the physiological advantages already mentioned and demonstrated when using scalp FU grafts.

Manipulation of the grafts is technically easier due to the rigidity of their structure (given by the elements mentioned that make up the FU). For this reason, the insertion of the graft with delicate forceps into the wound bed is facilitated.

FU grafts have a greater volume of subcutaneous tissue (located around the follicles of terminal hairs at a depth of 4 to 5 mm), which not only allows for the possibility of adding volume to the ulcer bed but also provides other non-follicular adult stem cells (e.g., stem cells derived from adipose tissue), thus increasing the healing response.^35^

Based on the premise that wounds heal faster when located in the skin with capillary follicles in the anagen phase rather than the telogen phase, the “theoretical” advantage of FU grafts follows. Since most of the hair follicles on the scalp (approximately 85% in the occipital region) are in the anagen phase, it is not only ideal as a donor area because punch incisions heal faster, but also because these anagenic hairs increase the healing response in the recipient wound.^18^

**Figure 7:**
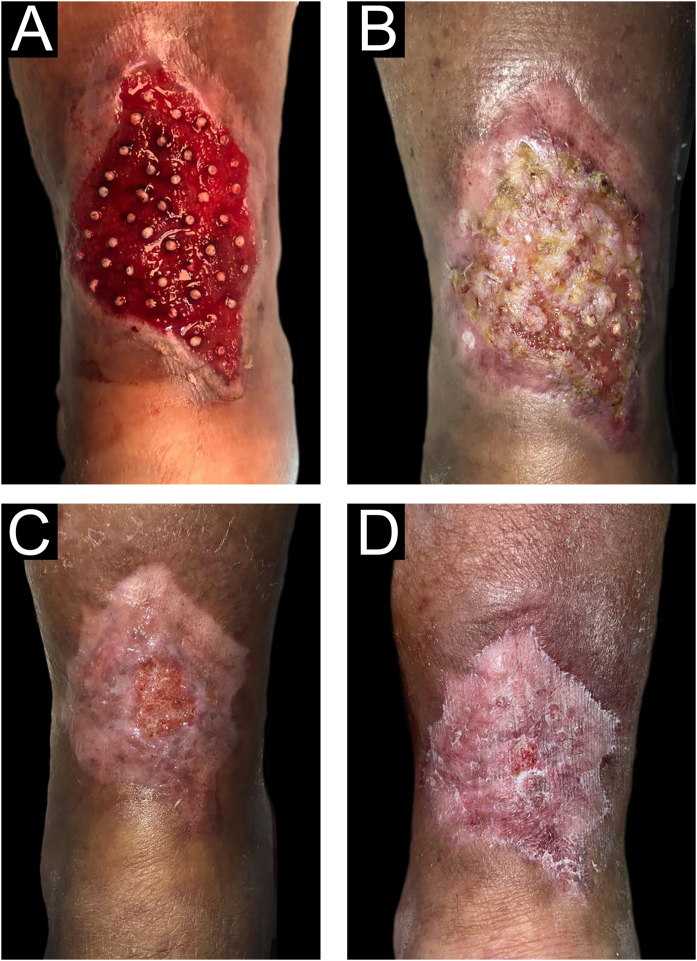
Sequence of healing process after follicular units (FU) graft on the leg. Medical appointment days: (A) 06/10/2021, (B) 07/15/2021, (C) 10/17/2021, (D) 11/11/2021.

The choice of the density of FU grafted into the ulcer bed is adapted to the present experience since the authors have found that higher density results in more popping or bouncing effects. The authors found that this density, although lower than that shown in the literature, also achieved a good rate of tissue regeneration (Fig. 7).

To implement this technique, a simple learning curve for the health care team is required, and it does not require complex or expensive technological equipment, but it does require an expert in the technique of FU harvesting who can transmit their expertise. As an observation, the authors could mention that a disadvantage is the time required to perform the technique, which could be laborious and prolonged based on the experience achieved.

## Conclusions

The wounds that will benefit from FU skin grafting are those that are in the last phase of healing, the epithelialization stage, of any etiology, and have adequate control of their underlying pathology, bacterial load, and pain, thus shortening healing times.

Skin grafting with FU is a simple surgical procedure that has so far demonstrated better results than conventional grafting techniques, benefiting healing due to the stimulus provided by bulge stem cells and leptin release from the anagen phase. It reduces perioperative and post-surgical complications due to less trauma in the donor and recipient areas, with lower intraoperative bleeding, lower infection rates, and less postoperative pain. The procedure is performed under local anesthesia and shows the rapid recovery of the donor area accompanied by simple postoperative care.

In addition, it is an outpatient surgical procedure that does not require hospitalization, resulting in a cost-effective practice.

The authors have good results with this technique, however more controlled and randomized studies with long follow-up are needed to assess efficacy, safety, and recurrences.

## Financial support

None declared.

## Author’s contribution

Anahi Belatti: Head of the Wound Care Section at the Dermatology department. Pushed forward the original idea for the academic and practical training of the surgical team and initiated the creation of hospital financing codes to be approved for external patients. Selected the surgical team.

Florencia Bertarini: Dermatologist, member of the surgical team, lead author of the article, reviewer of the surgical technique bibliography and composition of images and videos.

Virginia Pombo: Dermatologist surgeon, full member of the surgical team developing the technique.

Luis Mazzuoccolo: Head of the Dermatology department, who approved the development of the surgical technique for its implementation in the hospital and performed the article review.

Damian Ferrario: Dermatologist surgeon who participated in the training of the medical team for the development and implementation of the surgical technique, supporting the learning curve.

## Conflicts of interest

None declared.
