# Supplementary material for: Follicular unit grafting in chronic ulcers: a valuable technique for integrated management
Source: An Bras Dermatol. 2024 Mar 22;99(4):568–77. doi: 10.1016/j.abd.2023.08.012 (PMC11220926; doi:10.1016/j.abd.2023.08.012)
Supplement: Supplementary file 1 [file mmc1.docx]

**ABD-D-23-00334_Supplementary Material**

**Video 1** Anesthetic infiltration video.

**Video 2** Tumescent anesthetic infiltration of the scalp.

**Video 3** harvesting scalp FUs technique.

**Video 4** Graft technique (#1).

**Video 5** One-step technique (#2).

**Video 6** Stick and place.
